# The regulatory mechanism and potential application of IL-23 in autoimmune diseases

**DOI:** 10.3389/fphar.2022.982238

**Published:** 2022-09-13

**Authors:** De-Kai Xiong, Xiang Shi, Miao-Miao Han, Xing-Min Zhang, Na-Na Wu, Xiu-Yue Sheng, Ji-Nian Wang

**Affiliations:** ^1^ School of Health Management, Anhui Medical University, Hefei, China; ^2^ Department of Education, The First Affiliated Hospital of Anhui Medical University, Hefei, China

**Keywords:** IL-23, IL-23R, IL-23/TH17 axis, autoimmune diseases, RA

## Abstract

IL-23 is a heterodimeric pro-inflammatory cytokine secreted by dendritic cells and macrophages that belongs to the IL-12 family. It has pro-inflammatory effects and is a key cytokine and upstream regulatory cytokine involved in protective immune responses, stimulating the differentiation and proliferation of downstream effectors such as Th17 cells. It is expressed in various autoimmune diseases such as psoriasis, systemic lupus erythematosus (SLE), rheumatoid arthritis (RA). The IL-23/TH17 axis formed by IL-23 and TH17 has been confirmed to participate in autoimmune diseases pathogenesis. IL-23R is the receptor for IL-23 and plays an activating role. Targeting IL-23 is currently the main strategy for the treatment of various autoimmune diseases. In this review we summarized the mechanism of action and clinical application potential of IL-23 in autoimmune diseases by summarizing the latest research results and reviewing the literature, which would help to further understand IL-23 and provide a theoretical basis for future clinical targeting and drug development.

## Introduction

Autoimmune diseases (AD) are immunopathological states in which the body’s autoimmune tolerance mechanisms are dysregulated or disrupted, leading to damage or functional abnormalities in its own tissues and organs, and are considered to be a heterogeneous group of “common complex diseases.” It evolved from a complex interaction between the immune system and autoantigens, involving multiple genetic attributes, environmental triggers, and multiple cell types (da Costa et al., 2019). AD was first identified as a rare disease, but statistics have found that it affects 3%–5% of the population worldwide (JONES et al., 2007), rising to 5%–8% by 2020 (Fugger et al., 2020). According to the location of the lesion, AD is generally divided into two categories (Gao et al., 2021), organ-specific AD, such as myasthenia gravis (MG) with neurological involvement, and systemic AD, such as systemic lupus erythematosus (SLE). Genetic factors (Jonkers and Wijmenga, 2017) (including epigenetic (Jeffries, 2018), monogenic and polygenic inheritance (CANDORE et al., 2002)) and environmental factors (Cui et al., 2013) are considered to be common pathogenic factors in AD. Patients with AD usually have no obvious symptoms in the early stages of the disease, but there are distinctive features in the developmental stages of the disease, such as abnormal development and dysfunction of immune cells, production of large amounts of cytokines, disturbance of immune regulation, and disruption of T-cell homeostasis (Qian et al., 2022). The production and changes of immune cells and inflammatory cytokines may promote the development of autoimmune diseases and lead to persistent inflammation and tissue damage (Sumida et al., 2018). Currently, with the development of science and technology, some new immune cytokines are receiving more and more attention (Han et al., 2021).

IL-23 is a heterodimeric pro-inflammatory cytokine secreted by dendritic cells and macrophages that belongs to the IL-12 family (McKenzie et al., 2006). It is pro-inflammatory and can stimulate the differentiation and proliferation of downstream effectors such as Th17 cells and is expressed in a variety of diseases, such as tumors (Yan et al., 2018), periodontal disease (Bunte and Beikler, 2019), ocular diseases (Cui et al., 2022), and auto immune diseases, among others. Since its discovery, it has received widespread attention because it affects phagocytes and lymphocytes, has autocrine effects on macrophages (Cua et al., 2003) and dendritic cells (Belladonna et al., 2002), links innate and adaptive cellular immunity, it can also promote the occurrence of auto-inflammation and induce the proliferation and changes of tumor cells (Chan et al., 2014). IL-23R is the receptor for IL-23 and plays an activating role in AD. Targeting IL-23 and IL-23R remains an effective strategy for treating autoimmune diseases today.

An increasing number of studies have found that IL-23 is involved in the pathogenesis of AD. In this review we summarized the mechanism of action and clinical application potential of IL-23 in AD by searching PubMed and Web of Science electronic databases systematically without restricting the languages and years (up to 2022). Search terms included “IL-23” combined with “autoimmune diseases” or “Psoriasis” or “systemic lupus erythematosus (SLE)” or “rheumatoid arthritis (RA)” or “inflammatory bowel disease (IBD)” or “ankylosing spondylitis (AS)” or “nucleic acid aptamer” or “genome editing.” This review mainly elaborates the regulatory mechanism of action and clinical application potential of IL-23 in AD, which would help to further understand IL-23 and provide a theoretical basis for future clinical targeting and drug development.

### Overview of IL-23 and IL-23R

IL-23, belongs to the IL-12 cytokine family, is a heterodimeric pro-inflammatory cytokine. It is composed of a p40 subunit shared with IL-12 and its own unique p19 subunit (Tang et al., 2012) linked by disulfide bonds. The gene on human chromosome 5 encodes the p40 subunit, while the gene encoding the p19 subunit is located on human chromosome 12 (Kleinschek et al., 2006). IL-23p40 is a glycosylated type I soluble protein with a molecular weight of 34.7 kDa (Gubler et al., 1991), while IL-23p19 is a non-glycosylated protein with a molecular weight of 18.7 kDa (Oppmann et al., 2000).

IL-23 is mainly produced by dendritic cells and macrophages (McKenzie et al., 2006), but non-immune cells such as keratinocytes and synoviocytes can as well secrete it. There are also some immune receptors that enhance the production of IL-23, e.g., Gram-positive bacteria release peptidoglycan, which binds to the toll-like receptor (TLR) 4 (Re and Strominger, 2001) and Gram-negative bacteria produce lipopolysaccharide (LPS), which binds to the TLR3 receptor (Smits et al., 2004), which can effectively induce IL-23 secretion. There are several cytokines involved in the regulation of IL-23, e.g., TNF-α can increase IL-23 expression in fibroblast-like synoviocytes (FLs) (Goldberg et al., 2009), while TNF-α receptor 1 (TNFR1), which has hair-raising effects on macrophages and DC cells, can decrease IL-23 expression by downregulating p40 (Zakharova and Ziegler, 2005). In addition, the cytokine IL-10 has an anti-inflammatory effect and can also decrease IL-23 expression (Liu et al., 2009).

IL-23R is the receptor for IL-23, which is a heterodimeric structure composed of a heterodimer with the IL-12Rβ1 subunit and its own unique IL-23R subunit, located on human chromosome 19 encoding the gene that forms the IL-12Rβ1 subunit and on human chromosome 1 encoding the gene that forms the IL-23R subunit (Zhu et al., 2016). IL-12Rβ1 subunit is mainly expressed on T cells, monocytes/macrophages, natural killer (NK) T cells, and DC cells (Pahan and Jana, 2009), whereas IL-23 subunit is expressed on specific T cells (Gaffen et al., 2014) and minor expression on B cells and lymphoid cells (Chognard et al., 2014).

Studies have shown that IL-23 has pro-inflammatory effects and is a key cytokine (Curtis and Way, 2009) and upstream regulatory cytokine (Aggarwal et al., 2003) that can participate in protective immune responses to bacterial and fungal infections. The combined effect of IL-23 and IL-2 leads to a significant decrease in IgG and a significant increase in IgM in humans, indicating the critical composition of IL-23 in the primary immune response (Cocco et al., 2011). In addition, IL-23 promotes T cell proliferation, stimulates the differentiation and proliferation of downstream effectors such as Th17 cells (Stritesky et al., 2008), and induces the production of IL-17 (Li et al., 2016), which has been shown to be a major pro-inflammatory cytokine and a major medium in the pathogenesis of inflammatory and autoimmune diseases (Amatya et al., 2017), thus also demonstrating a role for IL-23 in autoimmune diseases.

### Function of IL-23 in autoimmune diseases

**FIGURE 1:**
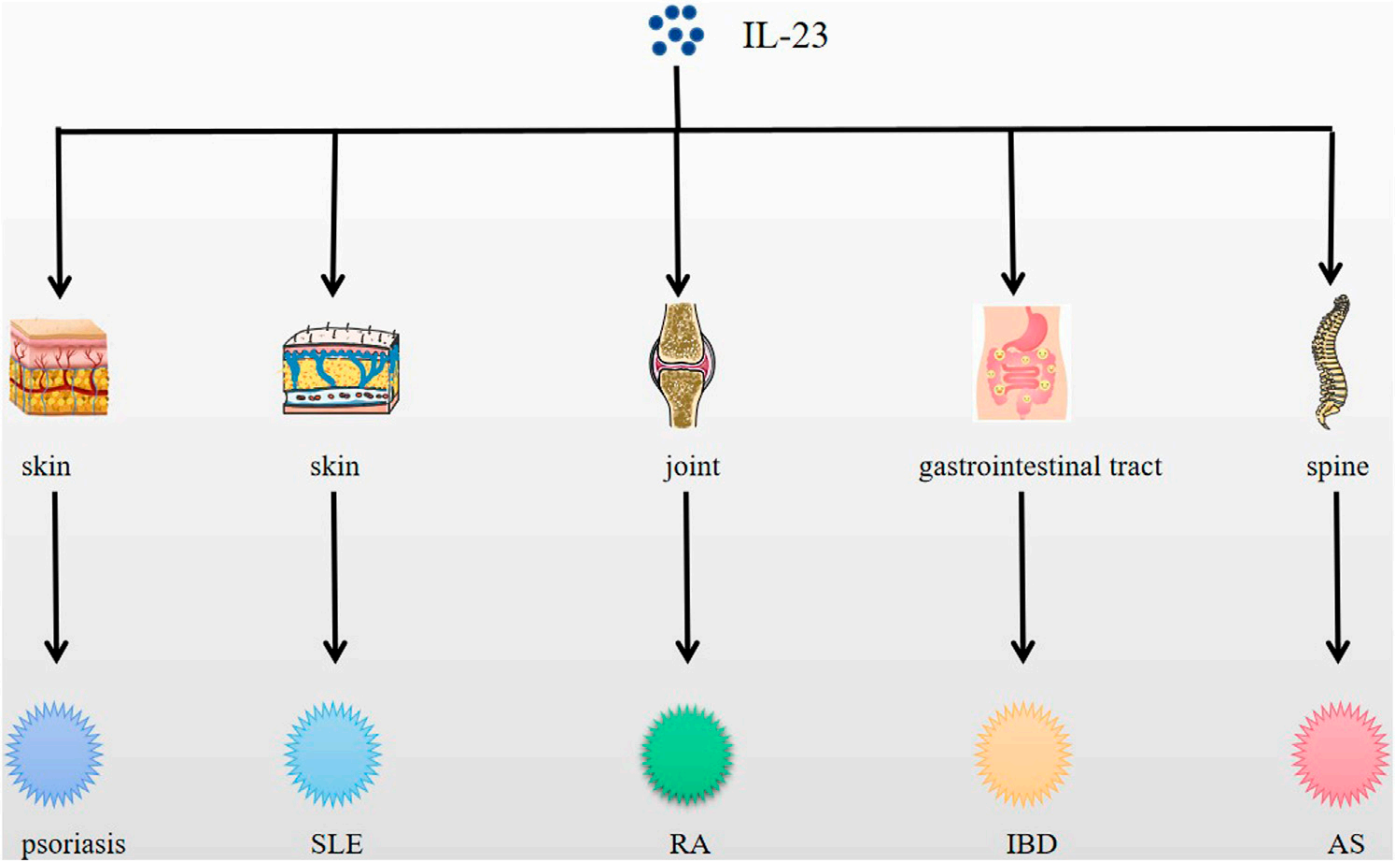
Expression of IL-23 in psoriasis, SLE, RA, IBD, AS and other diseases. IL-23 is expressed in skin, joint, spine, etc., and causes related autoimmune diseases, such as psoriasis, SLE, RA, AS, etc.

IL-23 functions in immune cells, affecting phagocytes and lymphocytes, and exerts autocrine effects on macrophages and dendritic cells, which in turn are closely related to autoimmune diseases (Singh et al., 2012), as a result, IL-23 plays a critical role in many autoimmune diseases development (Fragoulis et al., 2016) acting as a pro-inflammatory mediator and is expressed in a variety of autoimmune diseases like psoriasis, SLE, RA, IBD, etc, and differently (Tables 1–3) (Figure 1).

**TABLE 1:** Expression and function of IL-23 in autoimmune diseases.

Autoimmune diseases	Expression level of IL-23 in serum/plasma (PMID)	Function role of IL-23	References
Psoriasis	Human (+)	IL-23 stimulates Th17 cells to produce IL-17 and IL-22	28165883
		IL-23 is the main upstream regulator of psoriasis	14707118
SLE	Human (+)	IL-23 increased the ratio of IL-17 to IFN-c	20513356
RA	Human (+)	IL-23 influences T cell phenotype in SLE	22012611
IBD	Human (+)	IL-23 Promote and enhance the secretion of IL-17 by TH17 cells	29441874
AS	Human (+)	IL-23 stimulates the transformation of CD4^+^ T cells into Th17 cells	25288779

**TABLE 2:** The expression level of IL-23 in autoimmune disease tissues (clinical research).

Autoimmune diseases	The expression level of IL-23 in tissues	Subjects	References (PMID)
Psoriasis	Skin (+)	Human (+)	17074928
RA	Synovium (+)	Human (+)	22012611
IBD	Intestinal tissue (+)	Human (+)	21227898
AS	Marrow (+)	Human (+)	23508523

**TABLE 3:** The expression level of IL-23 in autoimmune disease tissues (pre-clinical research).

Autoimmune diseases	The expression level of IL-23 in tissues	Subjects	References (PMID)
Psoriasis	Skin (+)	Mice (+)	14707118
RA	Synovium (+)	Mice (+)	14662908
IBD	Mucosa (+)	Mice (+)	7595199

### Psoriasis

Psoriasis is an immune-mediated chronic inflammatory skin disease (Greb et al., 2016), and as a systemic disease, the mainly features are epidermal hyperplasia, skin changes, and dermal inflammatory cell infiltration (Armstrong and Read, 2020). Approximately 2%–3% of the global population suffers from psoriasis (Schonmann et al., 2019), which severely affects the quality of life of patients. The pathogenesis of the disease is also multifactorial, including ethnicity, geography, environment, genetics, and lifestyle (Huerta et al., 2007).

The pathogenesis of psoriasis is not yet fully understood, and immune cell dysfunction and abnormal expression of related cytokine levels are the main features of its pathogenesis. Studies have shown that the pathogenesis of psoriasis is related to cytokines such as IL-23 and IL-17 (Wang et al., 2020), and a large number of IL-17-producing lymphocytes and the p40 and p19 subunits of IL-23 were detected in psoriatic plaques (Lee et al., 2004). Experiments by Xiao et al. (2018), also found a significant upregulation of IL-17 and IL-23 compared with healthy controls in patients with psoriasis vulgaris. Psoriasis lesions are marked by infiltration of immune cells in the dermis, such as macrophages, dendritic cells and neutrophils (Sabat et al., 2019), and macrophages and dendritic cells secrete IL-23, which in turn induces IL-17 production by CD4+T cells (Hansel et al., 2011), thus suggesting that IL-23 is the main upstream regulator leading to the development of psoriasis lesions. Moreover, in the mouse experiments of van der Fits et al. (2009), it was found that inflammation was completely blocked in mice lacking IL-23 in a mouse model of psoriasis induced by IMQ, suggesting that down-regulation of IL-23 is a protective factor in the treatment of psoriasis. In addition, studies in humans have shown that direct injection of IL-23 through the dermis induces epidermal hyperplasia common in psoriasis patients (CHAN et al., 2006), reinforcing that upregulation of IL-23 expression is likely to be a key marker for psoriasis diagnosis.

### Systemic lupus erythematosus

SLE is a chronic autoimmune connective tissue disease that develops mainly due to an imbalance between pro-inflammatory factors (e.g., IFN-g, TH17) and regulatory cytokines (e.g., IL-2) (Gatto et al., 2013), leading to the production of multiple antibodies, the formation and deposition of immune complexes, and the interaction between the two leading to organ and tissue damage (Leng et al., 2010), resulting in clinical manifestations such as fever, headache, rash, and kidney damage. There are significant regional differences in the prevalence of SLE, with a global prevalence of approximately 0–241 per 100,000 and a prevalence of 30–70 per 100,000 in mainland China (Frances et al., 2017), and an adjusted global prevalence of 50–100 per 100,000 for SLE in 2021 (Fanouriakis et al., 2021).

The role of cytokines in the pathogenesis of SLE has been demonstrated (Illei et al., 2004), in particular TH17 has been detected in SLE patients and is thought to be responsible for the development of local inflammation in lupus patients (Crispin et al., 2008). Recent experiments in mice found high IL-23 levels expressed in SLE susceptible mice (Zhang et al., 2009) which suggests that IL-23 is also involved in its pathogenesis. In addition, IL-23 levels have also been found to be significantly elevated in the serum of SLE patients in human studies, such as Milena et al. (Vukelic et al., 2020) who measured serum levels of IL-23 in 56 subjects meeting the ACR SLE criteria by ELISA and showed that IL-23 levels were positively correlated with overall SLE disease activity as measured by SLEDAI, suggesting that IL-23 serum levels may be a possible biomarker for detecting SLE prevalence. First, IL-23 is able to differentiate T cells into pro-inflammatory cells TH17 (Korn et al., 2009), and second, IL-23 also has an effect on the T cell phenotype of SLE by inducing extrafollicular helper T cells (eTfh), driving B cell autoantibody production, while limiting IL-2 production and regulatory T cell (Treg) differentiation (Dai et al., 2017). To summarize, the regulation pathogenesis of SLE is affected by IL-23.

### Rheumatoid arthritis

RA is an autoimmune disease that leads to chronic joint inflammation disease and bone destruction, which is mainly characterized by systemic inflammation, articular cartilage, persistent synovitis and progressive joint destruction (Li et al., 2010), resulting in structural damage, functional disability and decline (Bingham, 2002). The preclinical manifestations of RA patients are pain or stiffness of joints with weakness, fatigue or anorexia (Rindfleisch and Muller, 2005). According to data, the global prevalence of RA is around 1% in 2021 (Hansildaar et al., 2021), and the prevalence is higher in women than in men, 2–3 times higher than in men (Salgado and Maneiro, 2014). It is generally believed that the pathogenesis of RA is related to genetic, infectious, and sex hormone factors, but it has also been shown that cytokines play a fundamental role in causing RA-related inflammation, joint destruction, and extra-articular manifestations (Brennan and McInnes, 2008), and RA synovitis is characterized by inflammatory infiltration and a synovial environment dominated by pro-inflammatory cytokines and chemokines (McInnes and Schett, 2007), in which IL-23, as a pro-inflammatory cytokine, is involved.

In a study by Craig et al. (Murphy et al., 2003) in an experimental RA mouse model, mice lacking IL-23p19 were found to be resistant and protective against arthritis induced by collagen, and mice targeting IL-23 did not show clinical signs of RA. In addition, studies by Rong et al. (2012) showing increased expression of IL-23 in synovial fibroblasts and plasma of RA patients, as well as Rasmussen et al. (2010) who found significantly higher plasma IL-23 levels in RA patients by performing a comparative study between RA patients and healthy controls, showed a correlation between IL-23 levels and RA activity and supported the hypothesis that IL-23 is a risk factor for RA. In fact, IL-23 can stimulate the proliferation and activation of TH17 in a pro-inflammatory state, and activated TH17 cells release inflammatory cytokines such as IL-17A (Paradowska-Gorycka et al., 2010). IL-17A can accelerate the proliferation and differentiation of osteoclasts (Hashizume et al., 2008), which can cause damage to joints and bone (Steinman, 2007). Therefore, the interaction between IL-23 and IL-17 is crucial for the pathogenesis of RA.

### Inflammatory bowel disease

IBD is a chronic inflammatory autoimmune disease of the gastrointestinal tract mediated by T lymphocytes and characterized by chronic intestinal inflammatory injury (Xavier and Podolsky, 2007), with the typical clinical presentation of abdominal pain, diarrhea and blood in the stool. It includes Crohn’s disease (CD), ulcerative colitis (UC) and indeterminate colitis (when overlapping features of CD and UC are observed) (Geremia and Jewell, 2012). The prevalence of IBD is higher in industrialized countries, such as Europe and the Americas (Loftus, 2004), and it is estimated that in 2020, there will be three million people with IBD in Europe, three million in the United States and more than 80,000 in Australia (Jakubczyk et al., 2020).

Studies have shown that the pathological mechanism of IBD is a chronic pathological inflammatory immune response in the intestinal lumen due to intestinal mucosal barrier damage and dysbiosis (Yoo and Donowitz, 2019), while its pathogenesis may be related to environmental, genetic, biological, and immunological causes. Indeed, many cytokines are also involved in the development and progression of chronic intestinal inflammation in IBD, for example, higher expression of IL-12 and TNF-a in patients with CD (Niessner and Volk, 1995); Increased IL-5 and IL-13 expression in UC patients (Heller et al., 2005); Increased expression of IL-23 observed in experimental models of colitis such as TNBS colitis and DSS colitis (Karaboga et al., 2017); in In experimental mouse TNBS model, the use of IL-23p40 antibody was effective in suppressing mucosal inflammation and pro-inflammatory cytokine production (Neurath et al., 1995). Similarly, IL-23 has been shown to be involved in the pathogenesis of IBD (Kim et al., 2007), and Zhu et al. (2017) used ELISA kits to detect serum levels of pediatric IBD patients, and detected the expression of IL-17A and IL-23 mRNAs; Liu et al. (2011) also found in their study that, compared to controls, adult IBD patients showed high expression of IL-23 mRNA protein and IL-17A levels in inflammatory bowel tissues *in vivo*, and further found that IL-23 significantly promoted the activation of intraepithelial lymphocytes and natural killer cells and their cytotoxicity in IBD patients, which caused inflammatory damage in a variety of tissues and organs (Wu and Wan, 2020). In fact, a p19 peptide-based vaccine for the treatment of colitis has been developed by a research group (Guan et al., 2013) and used in an experimental mouse TNBS model, which showed that the vaccine inhibited IL-23 production and reduced colitis activity, suggesting that IL-23 could be used as a target for the treatment of IBD.

### Ankylosing spondylitis

AS is a chronic inflammatory autoimmune disease associated with human leukocyte antigen B27 and is part of the spondyloarthritis group (SpA). The main manifestation of the disease is spinal stiffness and progressive ankylosis deformity, and as the disease progresses, the location of the lesions can involve the mesial bony joints, paravertebral soft tissues, and large peripheral joints, and even organs such as the eyes, lungs, kidneys, and cardiovascular system, eventually leading to functional impairment (Chatzikyriakidou et al., 2009). According to data, the prevalence of AS is around 0.09%–0.3%, and the prevalence is higher in males, and about 80% of AS patients will have their first symptoms before the age of 30 (Wang and Ward Michael, 2018).

The pathogenesis of AS may be related to the interaction of genetic, epigenetic and environmental factors, with the involvement of many cytokines (ZWIERS et al., 2012). In fact, the culprit in the pathogenesis of AS is the leukocyte antigen (HLA) B27, Peersen and Maksymowych (2019) and others mentioned in their article that HLA-B27, due to its unique biological and biochemical properties, leads to its ability to misfold, induce endoplasmic reticulum stress response, and produce folded protein response (UPR) (Colbert et al., 2010) and autophagy. In the rat experiments of DeLay et al. (2009), upregulation of the UPR gene was found to induce an increase in helper T cells (TH17) and the pro-inflammatory cytokines IL-17 and IL-23 as well as IFN-γ, suggesting that IL-23 plays a role in it. In a clinical study, Jansen et al. (2015), founded that AS patients expressed elevated levels of IL-17A and IL-23, which showed that IL-23 was pro-inflammatory in the pathogenesis of AS as well as promoting the proliferation and differentiation of osteoclasts. The presence of IL-23 expression in the terminal ileum of patients with subclinical intestinal disease AS (Ciccia et al., 2009) and in the cartilage bone marrow of patients (Appel et al., 2013) further confirmed the involvement of IL-23 in the pathogenesis of AS. In conclusion, IL-23 is indeed involved in AS pathogenesis, but the specific pathogenesis needs further study.

### Regulatory mechanism of IL-23 in autoimmune diseases

The pathogenesis of autoimmune diseases is inextricably linked to the regulatory mechanisms of signaling pathways (Banerjee et al., 2017), with different signaling pathways between different cells; for example, the NF-κB signaling pathway is associated with T cells and can also inhibit B cell differentiation and development (Blanchett et al., 2021). Similarly, IL-23 is involved in mediating through the IL-23/TH-17 axis, activating signaling pathways such as JaK-STAT, PI3K/Akt and NF-κB, which drive chronic inflammation and autoimmunity by promoting TH17 cells differentiation, thus contributing to autoimmune diseases pathogenesis (Gaffen et al., 2014).

**FIGURE 2:**
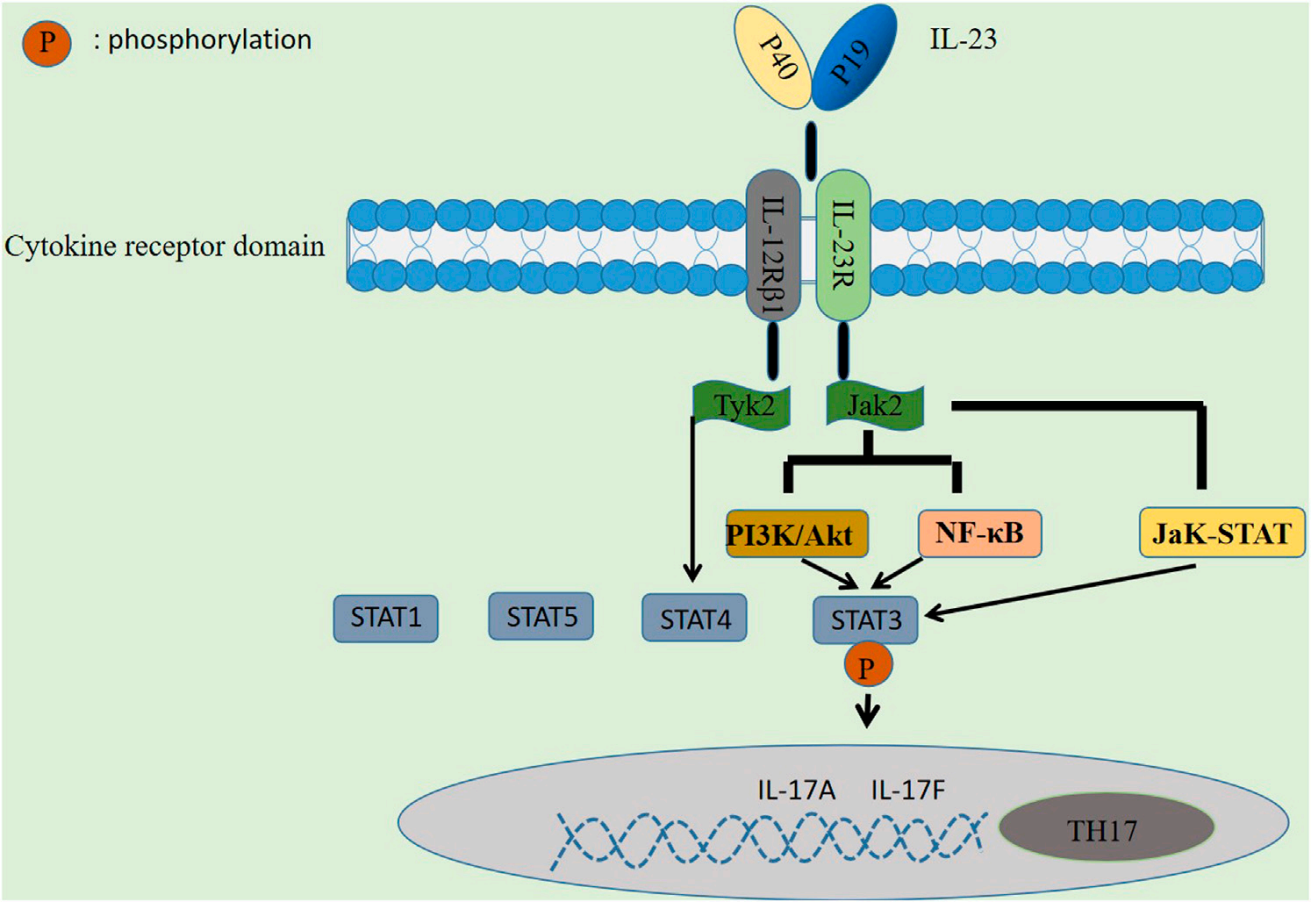
Regulatory mechanism of IL-23 in autoimmune diseases. IL-23 is involved in immune mediation by activating signaling pathways such as JaK-STAT, PI3K/Akt and NF-κB, thereby promoting the differentiation of TH17 cells to drive chronic inflammation and autoimmunity.

First, IL-23 activates the JaK-STAT signaling pathway, a signal transduction pathway composed of tyrosine kinase-related receptors, JaK kinase and STAT, activated by cytokines and their receptors (Bolli et al., 2003), which has been shown to be involved in cell proliferation, differentiation, survival, apoptosis, and autoimmune regulation (Koenders and Wimvan den Berg, 2015). It participates in the formation of skin lesions in a variety of autoimmune diseases and plays a role for initiating innate immunity, coordinating immunity mechanisms, and promoting anti-inflammatory immunity. There has been a great deal of research showing that the JaK-STAT signaling pathway plays an essential role in psoriasis (Sakkas and Bogdanos, 2017), SLE (Mathias and Stohl, 2020), RA (Malemud, 2018), and IBD (Banerjee et al., 2017). Four JaK proteins have been identified: JaK1, JaK2, JaK3, and TyK2, of which JaK2 and TyK2 are widely distributed and activated by IL-23 and its receptors in the human body. In this process, IL-23 binds to IL-23R and IL-12Rβ1 receptor complexes, respectively, and activates downstream JaK2 and TyK2, the receptor complexes generate phosphorylation reactions and the formation of STAT1, 3, 4, and 5 docking sites, which are then mediated by the involvement of activated JaK2 (Parham et al., 2002), followed by phosphorylation of STAT3 by docking, thereby promoting Th17 cell-specific transcription factor ROR-γt expression and enhance the level of IL-17 secretion by Th17 cells, and finally synthesize IL-17A, IL-17F and other inflammatory factors, as shown in Figure 2.

Second, in addition to the JaK-STAT signaling pathway, it has been shown that signaling pathways such as PI3K/Akt and NF-κB may also be involved in the regulation of AD by IL-23 (Watford et al., 2004). PI3K/Akt has been well demonstrated to be a signaling pathway associated with cell proliferation (Jones et al., 2005) and is widely expressed in a variety of cell types. Cho et al. (2006) found in mouse experiments that IL-23 binding to its receptor activates JaK2 protein, which in turn activates PI3K/Akt and NF-κB signaling pathways, and the activated PI3K/Akt pathway can also directly stimulate STAT3, resulting in a phosphorylation response that induces IL-17 production. In a study by Kim et al. (2005), they found that an increase in IL-17 expression in activated T cells in RA patients may be a consequence of the activation of PI3K/Akt and NF-κB signaling pathways.

In summary, although there is no clear research evidence, the above studies have sufficiently demonstrated that the possibility of IL-23 being involved in activating PI3K/Akt and NF-κB signaling pathways is very high, and if it is clear that IL-23 can activate PI3K/Akt and NF-κB signaling pathways and be involved in the regulation of AD, it will provide new ideas for future targeted therapies.

### Potential of IL-23 in clinical applications

Summarizing the above studies, we found that IL-23 is an upstream factor in the development of AD (e.g., SLE, IBD, RA, psoriasis, etc.), and induces the proliferation of psoriatic inflammation, the formation of RA osteoclasts by stimulating the proliferation and differentiation of IL-17 (Abdo and Tye, 2020), etc. And, in a study by Rasmussen et al. (2010), it has been shown that the plasma levels of IL-23 are significantly elevated in patients with early RA, indicating that IL-23 can be used for the purpose of a biomarker of early RA inflammation; serum IL-23 levels have also been shown to be positively correlated with RA disease activity (*r* = 0.697, *p* = 0.004) (Melis et al., 2010). Mirsattari et al. (2012) confirmed that there is a positive correlation between serum IL-23 levels and ulcerative colitis (UC) (*r* = 0.27, *p* = 0.04) and directly related to the severity of the disease (mean IL-23 serum levels in patients with mild UC = 296.2 ± 51.2 pg/ml; moderate = 356.1 ± 142.9 pg/ml; severe = 399.3 ± 163.8 pg/ml, *p* = 0.04), putting out that IL-23 may become the marker of disease diagnosis. In conclusion, both serum or plasma IL-23 levels have been shown to be associated with autoimmune disease pathogenesis and may provide a clinical basis for early detection, early treatment, and dynamic assessment of patient outcomes in AD patients.

In addition, in terms of clinical drugs, IL-23 can potentially be targeted to treat the symptoms of AD like SLE, psoriasis, IBD, and RA (Floss et al., 2015), preventing IL-23 from binding to its receptor has become a mechanism of current therapy, and IL-23 inhibitors have been shown to be effective (Han et al., 2020). The main IL-23 inhibitors that have been applied clinically are ustekinumab, guselkumab, and risankizumab.

Ustekinumab is the first FDA-approved biologic agent for psoriasis treatment that directly inhibits IL-23 (Tait Wojno et al., 2019). According to the clinical trial by Leonardi et al., 2008 and the long-term follow-up study by Papp et al., 2013 the experimental group treated with ustekinumab had higher PASI75 and PASI90 response rates than the control group treated with placebo. Treatment in the control group, demonstrating its effectiveness in the treatment of psoriasis. In a trial of ustekinumab in 20 patients with AS, it was observed that at week 24, 65% of patients achieved ASAS40 and 30% achieved partial remission of ASAS (Baraliakos et al., 2011), demonstrating the effectiveness of ustekinumab in the treatment of AS. Ustekinumab was approved as a treatment for Crohn’s disease (CD) in 2016 (Moschen et al., 2019). Thus, ustekinumab preparations can be used to treat a variety of autoimmune diseases.

Guselkumab is the first IL-23-specific inhibitor approved by the FDA to treat moderate-to-severe psoriasis (Megna et al., 2018). Sbidian et al. (2021)showed that guselkumab was more effective than ustekinumab, so in 2020 guselkumab was also approved by the European Medicines Agency (EMA) (Ghoreschi et al., 2021).

Risankizumab inhibitor has now completed two phase III studies (Reich et al., 2017) and is believed to be available in the near future.

**FIGURE 3:**
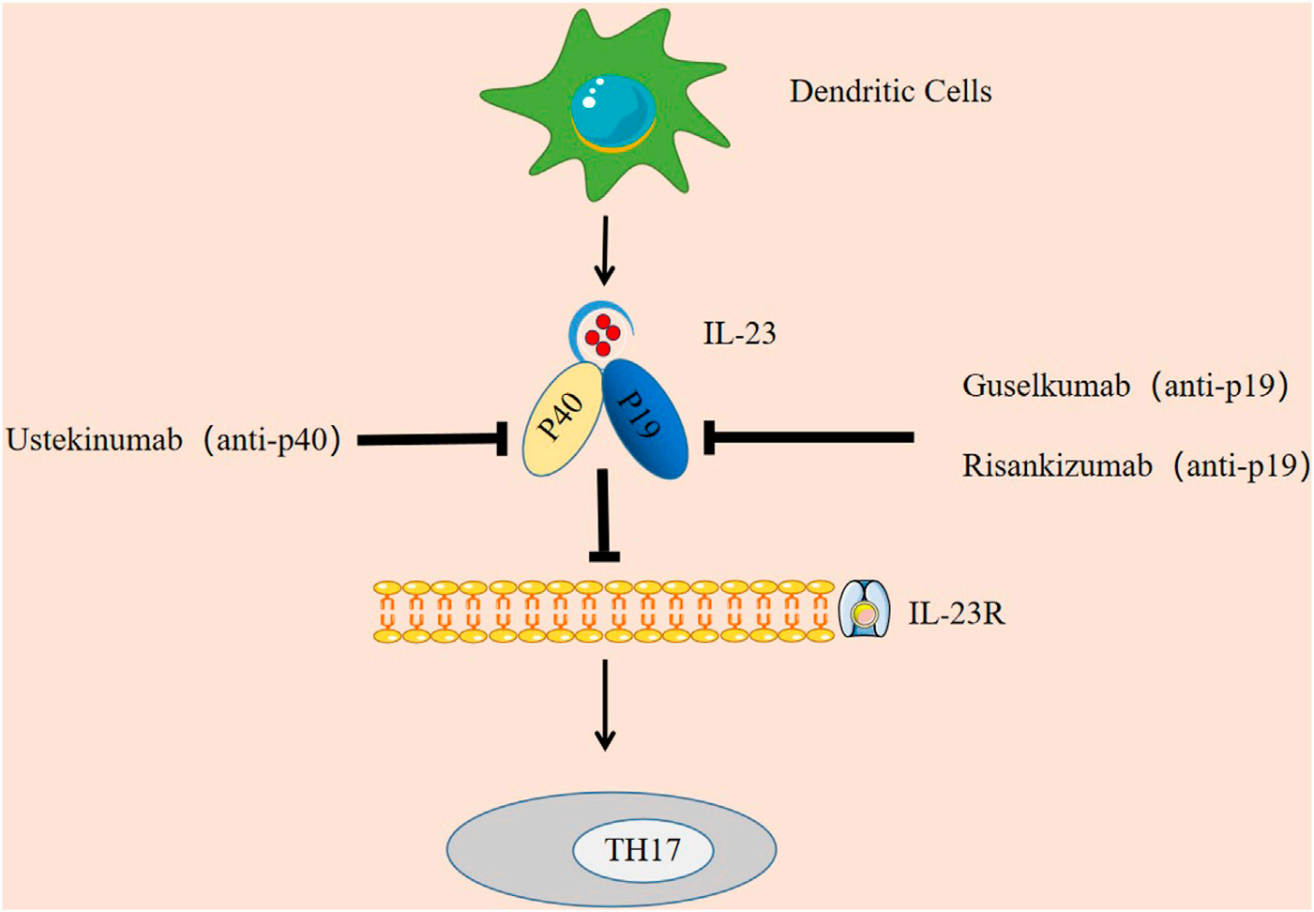
Targeting IL-23 in autoimmune diseases. IL-23 inhibitors have been shown to be effective. Ustekinumab is an inhibitor targeting IL-23p40, Guselkumab and Risankizumab are specific inhibitors targeting IL-23p19. They each block the IL-23 signaling pathway by binding to the p40 subunit and p19 subunit, thereby inhibiting the immune response of TH17.

In summary, the potential of IL-23 in clinical treatment is mainly manifested in two aspects. One is that IL-23 can be used as a diagnostic marker of disease, providing a basis for early detection and early treatment of patients. Another is drug therapy by targeting IL-23, such as IL-23 inhibitors (as shown in Figure 3).

### Future expectation

The current approach of anti-IL-23 treatment with biological agents is hardly enough to meet the current clinical treatment needs, and the medical community has started to explore new IL-23-based treatment techniques. For example: IL-23R-Fc fusion protein, nucleic acid aptamers, genome editing technology and the combined use of nanomaterials, all of which provide new ideas for future disease treatment.

IL-23R-Fc fusion protein, a new and reliable technique currently proposed in the medical community, allows the fusion of the extracellular structural domain (ECO) of IL-23R protein with the Fc fragment, possessing the ability to bind IL-23 while extending its serum half-life (up to 90 h) (Suen et al., 2010). Gao et al. (2019) tested the efficacy of this technique in alleviating PS and suppressing the inflammatory response through experiments in PS mice.

Nucleic acid aptamer, a new technology for specific molecular targeting using single-stranded DNA or RNA (Lakhin et al., 2013), has gradually entered the public perspective, and compared with antibodies, nucleic acid aptamer has advantages in cost, production difficulty and efficacy. At present there have been preclinical studies on aptamers for TNF-α, IL-6, and IL-17 in the medical community (Boshtam et al., 2017), Neil et al. (2017) have conducted an IL-23 aptamer study and successfully created the first IL-23 aptamer, which was delivered into human skin by topical administration and was shown to inhibit the mRNA levels of IL-17.

In addition, genome editing technology as a new technology is now widely used in treating autoimmune diseases (Mohammadzadeh et al., 2020), for example, by genome editing IL-37 can enhance mesenchymal stem cells (MSCs) thus providing a new and reliable approach for the treatment of SLE (Xu et al., 2020b). This offers new possibilities for IL-23 therapy, and in the future, altering IL-23 levels by genome editing techniques may also offer new approaches for the treatment of autoimmune diseases.

Furthermore, nanomaterials including environmental ultrafine particles (UFPs) and engineered nanoparticles (ENPs) (Pollard, 2020) have a wide range of roles in disease prevention and control as a relatively recent hot new technology in the scientific community (Zaheer et al., 2021). For example, it has been shown that IL-27 is able to inhibit multi-walled carbon nanotubes-induced TH17 cells (Moraes et al., 2013), thereby reducing the effects of EVE. This suggests that IL-23 can also be used in combination with nanotechnology to inhibit TH17 by reducing IL-23 activity levels to autoimmune diseases. In conclusion, IL-23 has unlimited potential in the treatment of autoimmune diseases, and new technologies and methods can provide new directions and options for future treatments.

## Conclusion

IL-23, a cytokine belongs to the IL-12 family, has common characteristics of IL-12 family cytokines and also interacts with TH17 cells due to its unique cellular structure, regulates each other and forms the IL-23/TH-17 axis that affects autoimmune diseases such as psoriasis, SLE, RA, etc. IL-23 is of interest for its unique ability to convert activated T cells into brain pro-inflammatory and potentially self-mutilating effector cells, and targeting IL-23 remains an effective strategy for treating autoimmune diseases today.

Of course, the regulatory mechanism of IL-23 in autoimmune diseases is not yet fully understood, and the exploration of new therapies for IL-23 needs to be further explored. It is believed that the mystery of IL-23 will be unveiled in the future research.
